# Somatostatin receptor expression in parathyroid neoplasms

**DOI:** 10.1530/EC-19-0260

**Published:** 2019-07-23

**Authors:** Sara Storvall, Helena Leijon, Eeva Ryhänen, Johanna Louhimo, Caj Haglund, Camilla Schalin-Jäntti, Johanna Arola

**Affiliations:** 1 Department of Endocrinology, Abdominal Center, University of Helsinki and Helsinki University Hospital, Helsinki, Finland; 2 Department of Pathology and Huslab, University of Helsinki and Helsinki University Hospital, Helsinki, Finland; 3 Department of Surgery, University of Helsinki and Helsinki University Hospital, Helsinki, Finland

**Keywords:** parathyroid, cancer, somatostatin, immunohistochemistry, hyperparathyroidism, tumor

## Abstract

**Introduction:**

Parathyroid carcinoma represents a rare cause of primary hyperparathyroidism. Distinguishing carcinoma from the benign tumors underlying primary hyperparathyroidism remains challenging. The diagnostic criteria for parathyroid carcinoma are local and/or metastatic spreading. Atypical parathyroid adenomas share other histological features with carcinomas but lack invasive growth. Somatostatin receptors are commonly expressed in different neuroendocrine tumors, but whether this also holds for parathyroid tumors remains unknown.

**Aim:**

Our aim is to examine the immunohistochemical expression of somatostatin receptor 1–5 in parathyroid typical adenomas, atypical adenomas and carcinomas.

**Methods:**

We used a tissue microarray construct from a nationwide cohort of parathyroid carcinomas (*n* = 32), age- and gender-matched typical parathyroid adenomas (*n* = 72) and atypical parathyroid adenomas (*n* = 27) for immunohistochemistry of somatostatin receptor subtypes 1–5. We separately assessed cytoplasmic, membrane and nuclear expression and also investigated the associations with histological, biochemical and clinical characteristics.

**Results:**

All parathyroid tumor subgroups expressed somatostatin receptors, although membrane expression appeared negligible. Except for somatostatin receptor 1, expression patterns differed between the three tumor types. Adenomas exhibited the weakest and carcinomas the strongest expression of somatostatin receptor 2, 3, 4 and 5. We observed the largest difference for cytoplasmic somatostatin receptor 5 expression.

**Conclusions:**

Parathyroid adenomas, atypical adenomas and carcinomas all express somatostatin receptor subtypes 1–5. Somatostatin receptor 5 may serve as a potential tumor marker for malignancy. Studies exploring the role of somatostatin receptor imaging and receptor-specific therapies in patients with parathyroid carcinomas are needed.

## Introduction

Primary hyperparathyroidism (PHPT) is a common endocrine disease usually caused by a parathyroid adenoma (in 85% of cases) or by glandular hyperplasia (15% of cases). Parathyroid carcinoma is only rarely the underlying cause (PC; about ~1% of PHPT). Symptoms of PC are related to the high serum calcium caused by the increased parathormone production and are the same but often more severe than those accompanying benign forms of PHPT. Furthermore, PC is very rare and difficult to diagnose, with no specific evidence-based treatment available (1, 2, 3, 4). Radical surgery is the only known potential cure for PC, while recurrence affects 20–60% of patients (5, 6, 7, 8). Furthermore, recent studies reported an increasing incidence of PC in Australia, the USA and Europe (8, 9, 10), although the reasons for this increase remain unknown. The diagnostic criteria of PC are based on histological examination demonstrating invasive tumor growth or evidence of metastatic disease (11). Similarly, atypical adenomas (APAs) share some histopathological features with PC, such as a fibrous septae and intratumoral hemorrhage, although lacking invasive growth (11). Biochemically severe PHPT might raise the suspicion of malignant disease before surgery. However, there are no disease-specific preoperative markers for PC, and it is nearly impossible to render a preoperative diagnosis of PC if the patient has no signs of metastatic disease. Some immunohistochemical markers indicating aggressive tumor behavior in PC and APA exist, such as a high Ki-67 percentage (>5%) (2, 12). A negative parafibromin stain is present in up to 75% of sporadic PCs, but rarely in adenomas (13, 14). Parafibromin is encoded by *CDC73*, a tumor suppressor gene. Germline mutations of *CDC73* lead to the loss of parafibromin expression and are associated with a high risk of PC (15, 16). *CDC73* germline mutations underlie the hyperparathyroidism jaw-tumor syndrome (HPT-JT), characterized by early-onset PHPT, ossifying jaw fibromas, kidney and uterine tumors, but may also cause familial isolated hyperparathyroidism (15, 16, 17). Parafibromin immunohistochemistry is thus a useful marker. The specificity of negative staining is high (18) and prompts genetic studies, that is search for *CDC73* germline mutations and genetic counseling, as appropriate. However, there are exceptions and more than 20% of PC cases stain positive for parafibromin on immunohistochemistry.

Somatostatin receptors (SSTs) mediate the effects of the hormone somatostatin. These membrane-bound G-protein-coupled receptors mediate their effects by altering the levels of intracellular calcium and cAMP. They may also heterodimerize with each other, as well as with β-adrenergic and opioid receptors, which, depending on the receptor type, can inhibit or enhance the effects of SST. There are five known SST subtypes: SST_1_, SST_2_, SST_3_, SST_4_ and SST_5_. Through alternative splicing, two variants of SST_2_ (SST_2_a and SST_2_b) exist. Moreover, SSTs can be found throughout the body with differing subtype distributions in various organs and tissues: in the central nervous system, the gastrointestinal tract, the pancreas and the kidneys as well as in leukocytes, endothelial cells and macrophages (19).

Somatostatin analogs are widely used in the treatment of different neuroendocrine tumors, such as ACTH-producing pituitary adenomas, somatotropinomas and gastropancreatic neuroendocrine neoplasms. They not only suppress hormonal hypersecretion by binding to different SSTs, but also have an antiproliferative effect on tumor cells (20). The somatostatin analog octreotide as well as peptide receptor-based radiotherapy, both of which primarily target SST_2_, have been used in the treatment of PTH-related protein (PTHrP)-dependent hypercalcemia caused by metastatic gastroenteropancreatic neuroendocrine tumors. As such, diminished calcium and PTHrP concentrations as well as stable disease were achieved in some patients (21). A previous study found somatostatin receptors 1, 3 and 4 expression in normal parathyroid cells (22). No previous studies on the expression of SSTs in neoplastic parathyroid tissue appear in the literature.

Therefore, we aimed to investigate the expression profile of somatostatin receptor subtypes in different parathyroid tumors underlying PHPT in a large patient cohort. We also sought to determine whether SSTs could serve as tumor markers in the differential diagnosis of typical parathyroid adenomas, atypical adenomas and carcinomas, perhaps as additive PC immunohistochemistry panel markers to the previously established parafibromin, Ki-67, galectin 3 and protein gene product 9.5 (PGP9.5) (15).

## Materials and methods

### Tumor material and patient cohort

Our material comprised 132 tumor samples from three tissue subgroups: parathyroid carcinomas (PCs), atypical parathyroid adenomas (APAs) and parathyroid adenomas (PAs).

The PC group consisted of tissue specimens from all patients diagnosed with PC in Finland between 2000 and 2011 (*n* = 32) (8). The PC patients were identified from the Finnish Cancer Registry using the ICD-10 code (C75.0) and from the databases of the five Finnish university hospitals and eight Finnish central hospitals, including their pathology databases.

Both the APA patients (*n* = 27) and the PA patients (*n* = 72) were retrieved from the Helsinki University Hospital pathology database (HUSLAB). The APA group (*n* = 27) consisted of a consecutive cohort diagnosed between 2000 and 2011. The PA patients were age and gender matched with the PC group. All PHPT patients with APAs and PAs were treated in the Endocrine Department at the Helsinki University Hospital.

Clinical data as well as the basic immunohistochemical characteristics of the tumors were described previously (8). Briefly, six PC patients had metastatic disease and two had local recurrence. None of the patients had MEN1. More information on the PC group appears in Table 1.

**Table 1:** Characteristics of patients with parathyroid carcinoma. Numbers are presented as median (range).

Number of patients	32
Age at diagnosis (years)	64 (35–83)
Preoperative serum ionized calcium (mmol/L)	1.76 (1.38–2.58)
Preoperative PTH (ng/L)	989 (68–4000)
Ki-67%	5 (0–40)
Patients with metastatic disease or local recurrence	7
Disease-related death	5

The Ethics Committee of Helsinki University Hospital approved the study protocol. Permission to use the histological specimens for this study without requiring individual informed consent was granted by the Finnish National Supervisory Authority for Welfare and Health (Valvira) (Dnro 8031/06.01.03.01/2015).

### Tissue microarray

The tissue microarray (TMA) blocks were created as described previously. Briefly, the most representative tumor blocks were chosen and six 1.0 mm cores from PC and APA samples, and three cores for PAs were punched for the TMA blocks, representing both the tumor border and central areas (8).

### Immunohistochemistry

Tissue sections (3.5 μm) were cut using a microtome. Heat-induced antigen retrieval was performed after deparaffinization using xylene and graded alcohol series. Sections were incubated using primary antibodies. Table 2 summarizes information on the antibodies used as well as the staining details. These antibodies have previously been used by our research group also including negative controls (23), and their specificity have been confirmed by Western blot and *in vitro* receptor autoradiography (24, 25, 26, 27, 28). Antibody binding was visualized using the polymer-based OptiView and UltraView Universal DAB Detection Kit (Ventana Medical System, Inc., Tucson, AZ, USA) or the EnVision Detection Systems (Dako, Agilent Pathology Solutions). Automated (Benchmark Ultra, Ventana) or semi-automated (AutoStainer, Lab Vision Corp., Fremont, CA, USA) staining instruments were used. Mayer’s hematoxylin (Dako) was used for the counterstaining on all slides. In addition, intestinal mucosa and Langerhans’ islet cells were used as positive controls. Normal parathyroid tissue was stained using SST antibodies for comparison.

**Table 2:** Antibodies and staining protocols used.

Receptor	Clone	Epitope sequence	Antibody	Company	Dilution	Dilution time
SSTR1	UMB-7	ENLESGGVFRNGTCTSRITTL	Ab137083	Abcam	1:500	45 min
SSTR2	UMB-1	ETQRTLLNGDLQTSI	Ab134152	Abcam	1:300	32 min
SSTR3	UMB-5	QLLPQEASTGEKSSTMRISYL	Ab137026	Abcam	1:7000	60 min
SSTR4	Sstr4	CQQEALQPEPGRKRIPLT	MCA5922	AbD Serotec	1:500	30 min
SSTR5	UMB-4	QEATPPAHRAAANGLMQTSKL	Ab109495	Abcam	1:1000	30 min

### Scoring

**Figure 1:**
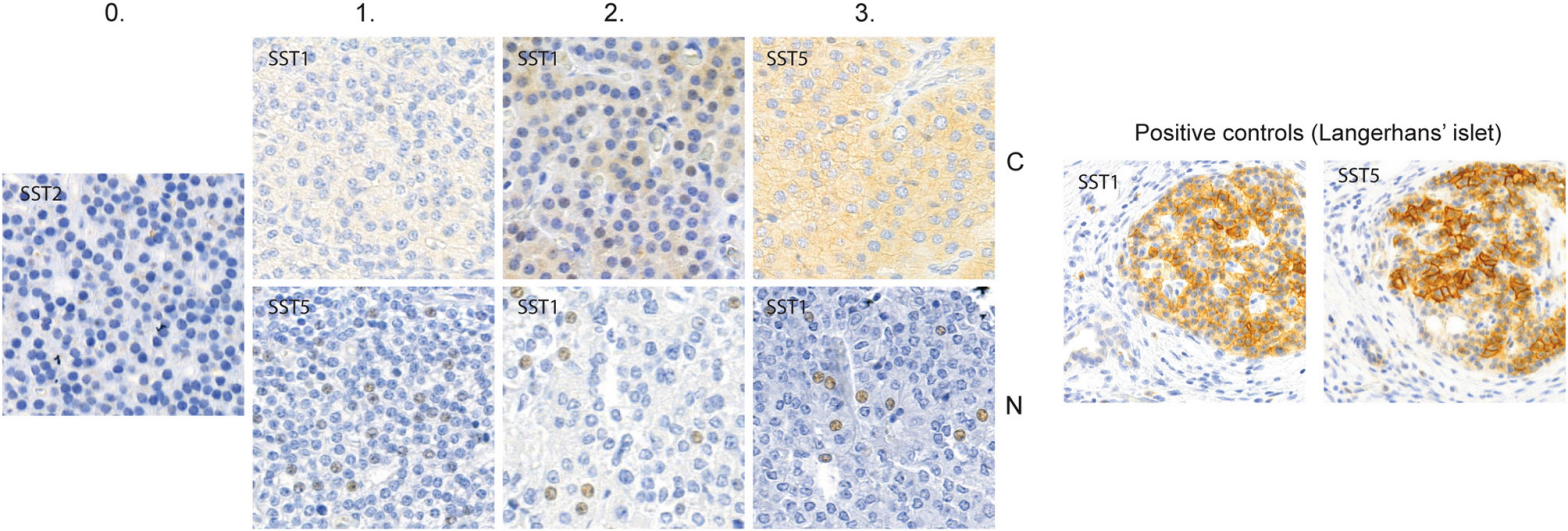
Examples of the staining intensities in parathyroid tumor tissue for the scoring system used in our study for cytoplasmic and nuclear expression. Membrane expression was weak and the intensity was not sufficiently diverse to make a similar comparison. Left: negative staining (0). Subsequent: weak cytoplasmic and nuclear staining (1), strong cytoplasmic and nuclear staining (2) and very strong cytoplasmic and nuclear staining (3). On the right are pancreatic islets stained with SST1 and SST5 presenting cytoplasmic and membrane positivity, functioning as positive control. Nuclear positivity is not found in the control stainings.

The immunohistochemical scoring was done independently by two researchers (SS and HL), without knowledge of the nature of the tumors. For any disagreements, the score was determined by reaching consensus following a discussion of the case. Staining was assessed separately for cytoplasmic (C), nuclear (N) and membranous (M) staining. Examples of representative SST stainings appear in Fig. 1. Staining was considered positive if at least 1% of the tumor cells per spot was stained. The intensity of the staining was estimated using a score of 0 to 3, where 0 represented completely negative and 3 represented as intense staining as in the positive control tissue. For each tumor, the TMA spot with the strongest staining intensity was used in the statistical analyses. Overall, the staining intensity was weak, and the intensity was not taken into account in the primary analyses, that is, TMA spots were only considered positive or negative. Scoring was carried out using 10× and 20× magnification for SST_2_, SST_3_ and SST_4_. The slides stained with antibodies for SST_1_ and SST_5_ were digitized using a panoramic scanner (3DHISTECH) and scoring was performed on digitized slides using the CaseViewer software (3DHISTECH), also relying on 10× and 20× magnifications.

### Statistical analysis

We used IBM SPSS Statistics version 23 (SPSS, Inc.) and RStudio version 1.0.153 (RStudio, Inc.) software for all statistical analyses and data processing. We considered *P* < 0.05 (two-tailed) as statistically significant. We used the χ^2^ test and the Fisher’s exact test, as appropriate, to investigate the relationships between categorical parameters. The Mann–Whitney *U* test was used for continuous variables.

## Results

**Figure 2:**
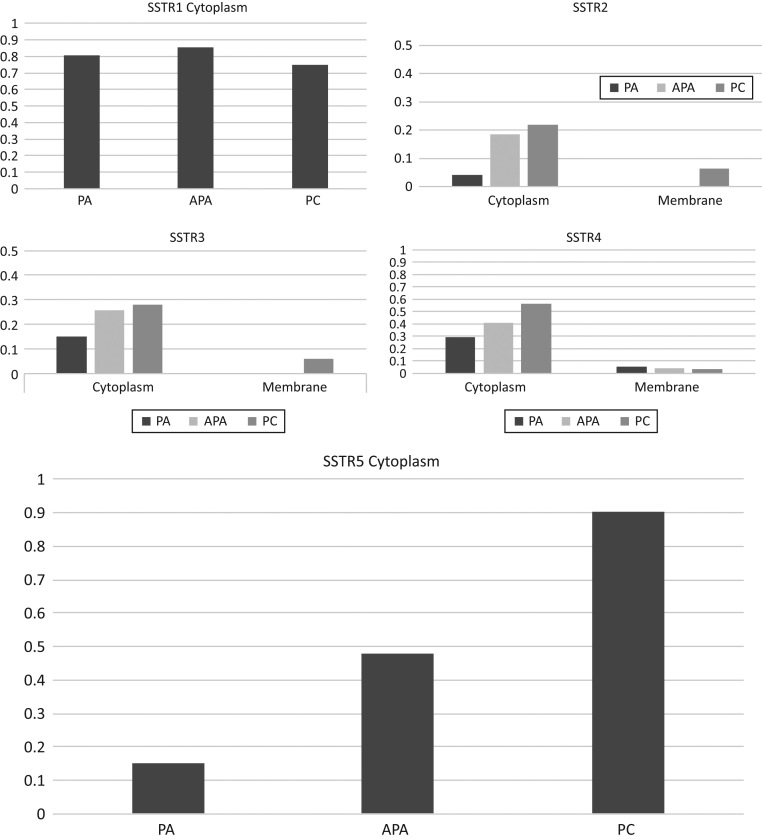
Distribution of the cytoplasmic and membranous SSTs in parathyroid carcinoma (PC), atypical parathyroid adenoma (APA) and benign parathyroid adenoma (PA). The Y axis represents the proportion of positively stained TMA spots.

All SST subtypes were to some degree expressed in parathyroid tumors. Normal parathyroid tissue (*n* = 6) was negative, except for some weak, diffuse cytoplasmic SST_5_ staining in four of six tissue samples. Cytoplasmic SST_1_ and SST_5_ were the most abundantly expressed SST subtypes in parathyroid tumors. Yet, membrane expression was negligible in all receptor subtypes and was, therefore, excluded from comparisons. Individual tumors, however, were of particular interest. Detailed information regarding SST expression appears in Fig. 2 and Table 3.

**Table 3:** Tumors staining positive for SSTR.

Receptor	PC	APA	PA	*P* value
SSTR1 C	13/32	9/27	30/72	0.512
SSTR1 N	17/32	18/27	50/72	0.269
SSTR2 C	7/32	5/27	3/72	0.01^a^
SSTR2 M	2/32	0	0	
SSTR3 C	9/32	7/27	11/72	0.264
SSTR3 N	9/32	6/27	2/72	<0.001^a^
SSTR3 M	2/32	0	0	
SSTR4 C	18/32	11/27	21/72	0.032^a^
SSTR4 N	20/32	14/27	23/72	0.009^a^
SSTR4 M	1/32	1/27	4/72	0.873
SSTR5 C	28/32	15/27	11/72	<0.001^a^
SSTR5 N	30/32	20/27	50/72	0.038^a^

^a^Significant *P* values.

### Somatostatin receptor 1 (SST_1_)

Membrane expression was absent for all tumor subtypes. Yet, both nuclear and cytoplasmic expression of SST_1_ was present, although expression did not differ significantly between the three tumor subgroups.

### Somatostatin receptor 2 (SST_2_)

Cytoplasmic staining was most prevalent in carcinomas and less so in APA and PA (*P* = 0.01). However, staining was scarce for all tumor subtypes. Interestingly, membrane positivity was found in only two carcinomas.

### Somatostatin receptor 3 (SST_3_)

Cytoplasmic expression was weak and did not differ between the tumor subgroups. As with SST_2_, we found two SST_3_ membrane-positive carcinomas. These were not the same tumors that demonstrated SST_2_ membrane positivity. The nuclear expression of SST_3_ in the PC group was increased, compared to the expression in the APA and PA subgroups (*P* < 0.001).

### Somatostatin receptor 4 (SST_4_)

Both cytoplasmic and nuclear expression increased in PC compared to the other tumor groups (cytoplasmic *P* = 0.032; nuclear *P* = 0.009). Membranous expression occurred in a small number of tumors across all tumor subgroups, although the difference between groups was not significant.

### Somatostatin receptor 5 (SST_5_)

The largest difference between groups was found in the expression of SST_5_. The cytoplasmic expression of SST_5_ appeared weakest in PAs, intermediate in APAs and highest in PC, since nearly all carcinomas were positive (*P* < 0.001). The positive predictive value (PPV) of positive cytoplasmic SST_5_ staining is 0.52, while the negative predictive value (NPV) of SST_5_ staining is 0.94. Furthermore, the nuclear expression differed significantly between subgroups (*P* = 0.043). Membrane SST_5_ staining was negative.

### Association with other parameters

Table 4 summarizes the correlations between SST expression and other biomarkers. Only three of our PC tumors had negative parafibromin staining, and another 12 had diminished staining. Negative parafibromin staining was associated with cytoplasmic SST_2_ (*P* = 0.04) and nuclear SST_3_ (*P* = 0.019) positivity. There was no association between expression of parafibromin and SST_5_ (*P* = 0.192).

**Table 4:** SSTR expression for all tumor groups in relation to Ki-67, tumor size, s-Ca-ion and s-PTH expressed as median (range).

	SSTR2C		
	Positive tumors	Negative tumors	*P* value
Ki-67 (%)	5.8 (0–30)	3.1 (0–40)	0.069
Tumor size (cm)	2.48 (1.0–4.0)	2.0 (1.0–5.0)	0.142
S-Ca-ion (mmol/L)	1.69 (1.38–2.42)	1.56 (1.28–2.58)	0.098
S-PTH (ng/l)	727 (34–2001)	514 (68–4000)	0.198
	**SSTR3N**		
	Positive tumors	Negative tumors	*P* value
Ki-67	5.6 (0–20)	3.1 (0–40)	0.003^a^
Tumor size (cm)	2.54 (1.0–4.0)	2.0 (1–5)	0.09
S-Ca-ion (mmol/L)	1.63 (1.32–2.02)	1.56 (1.28–2.58)	0.107
S-PTH (ng/L)	1049 (147–2769)	461 (34–4000)	0.003^a^
	**SSTR4C**		
	Positive tumors	Negative tumors	*P* value
Ki-67	4.1 (0–40)	3.1 (0–40)	0.048^a^
Tumor size (cm)	2.2 (1.0–5.0)	2.0 (1.0-5.0)	0.607
S-Ca-ion (mmol/L)	1.60 (1.29–2.58)	1.55 (128–2.42)	0.142
S-PTH (ng/L)	613 (68–4000)	485 (34–2220)	0.139
	**SSTR4N**		
	Positive tumors	Negative tumors	*P* value
Ki-67	4.1 (0.40)	3.0 (0–30)	0.225
Tumor size (cm)	2.23 (1–5)	2.0 (1.0–4.0)	0.825
S-Ca-ion (mmol/L)	1.57 (1.29–2.04)	1.58 (128–2.58)	0.487
S-PTH (ng/L)	580 (34–2220)	500 (68–4000)	0.258
	**SSTR5C**		
	Positive tumors	Negative tumors	*P* value
Ki-67	4.0 (0–40)	1.4 (0–30)	<0.001^a^
Tumor size (cm)	2.20 (1.0–5.0)	2.0 (1.0–5.0)	0.135
S-Ca-ion (mmol/L)	1.68 (1.32–2.58)	1.45 (1.28–2.42)	<0.001^a^
S-PTH (ng/L)	634 (34–4000)	173 (73–2210)	<0.001^a^
	**SSTR5N**		
	Positive tumors	Negative tumors	*P* value
Ki-67	0.0 (0–40)	0.0 (0–10)	0.267
Tumor size (cm)	2.0 (1.0–5.0)	2.0 (1.0–4.0)	0.079
S-Ca-ion (mmol/L)	1.52 (1.28–2.58)	1.45 (1.29–1.85)	0.141
S-PTH(ng/L)	264 (34–4000)	188 (77–2220)	0.230

^a^Significant *P* values.

A higher Ki-67 level was associated with the positive expression of cytoplasmic SST_4_ (*P* = 0.048) and SST_5_ (*P* < 0.001) as well as the nuclear expression of SST_3_ and SST_5_ (*P* = 0.003 and *P* < 0.001, respectively). Tumor size was not related to the expression of any of SST subtypes. Yet, high preoperative serum-ionized calcium and PTH concentrations were both associated with the expression of cytoplasmic SST_5_ (*P* < 0.001 for both).

### The role of somatostatin receptors in PCs

The expressions of SST_5_, SST_2_M and SST_3_M were examined in carcinomas exclusively. The expression patterns in relation to disease aggressiveness appear in Table 5.

**Table 5:** Expression of SST_5_C, SST_3_M and SST_2_M in relation to disease aggressiveness in PC.

	SST expression (number of tumors)		
	SST_5_C	SST_3_M	SST_2_M
Patients with recurrent/metastatic disease (*n* = 7)	5/7	1/7	1/7
Patients died from disease (also included in recurrent/metastatic, *n* = 5)	4/5	1/5	1/5
Patients with non-recurrent disease (*n* = 25)	23/25	1/25	1/25

We found that SST_5_C-negative carcinomas (*n* = 3) may be more aggressive than SST_5_C-positive carcinomas. One SST_5_C-negative PC patient died of disease, and another had metastatic disease. The third presented with non-recurrent disease. The median circulating PTH concentrations (1187 ng/L vs 823 ng/L) and Ki-67 (13 vs 7%) were higher in SST_5_C-negative PCs compared to SST_5_C-positive tumors, although this difference was not statistically significant (Mann–Whitney *U*).

We also examined membrane-positive tumors for SST_2_ and SST_3_ separately. One SST_3_M-positive PC patient died of disease. The other SST_3_M-positive tumor appeared SST_5_C-negative but was otherwise unremarkable. In addition, one SST_2_M-positive PC patient was also SST_5_C negative and died from the disease. The SST_2_M-positive tumor patients exhibited a slightly higher serum ionized calcium concentration than the remainder of the group (median 2.09 mg/L compared to 1.75 mg/L). The SST_2_M-positive tumor patients were also characterized by higher Ki-67 (2 vs 5%). All differences between SST_2_M-positive tumors and the remainder of the PC group were not statistically significant.

## Discussion

Here, we demonstrate that SSTs 1 through 5 are expressed in different parathyroid tumors, while SST_5_ is particularly abundant in PCs. Furthermore, we show that SST_5_ expression correlates with the tumor Ki-67 proliferation index, as well as with the circulating calcium and PTH concentrations. In addition, the expression of cytoplasmic SST_2_, SST_4_ and SST_5_ as well as nuclear SST_3_, SST_4_ and SST_5_ consistently mirror the lowest expressions in adenomas, the intermediate expression in atypical adenomas and the highest expression in carcinomas. While membrane positivity is very seldom detectable, four PCs showed membrane positivity either for SST_2_ or SST_3_, suggesting that SST_2_- and SST_3_-targeted imaging and therapies could potentially improve the management of these patients.

Taniyama *et al*. demonstrated the expression of SST_1_, SST_3_ and SST_4_ (and to some degree SST_5_) expression in chief cells of normal parathyroid tissue using immunohistochemistry relying on polyclonal antibodies (22). We used monoclonal antibodies, demonstrating scarce expression of SST_5_ in normal parathyroid cells; but, in contrast to Taniyama *et al*., we could not detect the expression of SST_1_, SST_3_ or SST_4_ in normal parathyroid tissue. To our knowledge, our study represents the first on SST expression in neoplastic parathyroid tissue. SST expression was lowest in adenomas and highest in carcinomas, with atypical adenomas falling in between. Negative SST stainings for normal parathyroid tissue fit with the pattern of an increased SST expression and an increasing neoplasticity.

The expression of somatostatin receptors carries a prognostic role in other neuroendocrine tumors. In gastroenteropancreatic NETs (GEP-NETs), SSTs are generally overexpressed in well-differentiated tumors with a decreasing expression in dedifferentiated tumors (29). In GEP-NETs, SST_2_ is the most frequently expressed receptor (30). The presence of SST_5_ and the absence of SST_2_ correlate with metastasis, angioinvasion and tumor growth (31). In pancreatic NETs, the low expression of SST_2_ and SST_5_ associates with a worse disease prognosis (32). When analyzing the PC group exclusively, we found that SST_5_C-negative carcinomas appeared to have higher calcium and PTH levels than the entire PC group average. These parameters previously investigated in our cohort strongly correlated with the nature of the tumor. Thus, the association between these parameters and SST expression is biased given that all these factors correlate with malignancy (8). Furthermore, the number of PCs with an adverse outcome is small.

The antibodies used in our study have been previously used and verified (25, 26, 27, 28, 33) with Western blotting and/or *in vitro* somatostatin receptor autoradiography. The same antibodies have previously been used by our research group for a study on pulmonary carcinoid tumors (23) as well as pheochromocytomas and paragangliomas (antibodies for SST_2_-SST_4_ (34)). We stained control tissue (pancreas and small intestine) to serve as a test for the functionality of the staining protocol as well as internal negative control. The Langerhans’ islet cells and enteroendocrine cells stained positive appropriate to the SST antibody used, while the surrounding tissue remained negative. Thus, one can assume that the staining we have observed is, in fact, valid also regarding the nuclear expression of SSTs.

In general, somatostatin receptors are considered membrane-bound G-protein-coupled receptors (35), with some degree of cytoplasmic staining on immunohistochemistry. We found that the membrane expression of SSTs were both faint and scarce. Ligand binding to somatostatin receptors induces receptor internalization by endocytosis for the lysosomal regrading or recycling of the receptor to the plasma membrane (36, 37). This could represent one mechanism explaining the cytoplasmic SST staining. Previous studies indicate that antibodies for SST_2_ generally stain the plasma membrane, while SST_1_, SST_3_ and SST_5_ stain the cytoplasm (38). Furthermore, SST expression generally decreases with a higher tumor grade as demonstrated in different NETs including pituitary adenomas, GEP-NETs, pheochromocytomas and paragangliomas, neuroblastoma, Merkel cell carcinoma and medullary thyroid carcinoma (38). Most previous studies have focused on membrane expression. We recently demonstrated that in pheochromocytomas and paragangliomas SST_1–5_ expression is either predominantly membranous (SST_2_) or cytoplasmic (SST_1_, _3–5_) (34). In the present study, nuclear and cytoplasmic SST expression appeared in the majority of parathyroid tumors, with significantly different expression profiles in adenomas, atypical adenomas and carcinomas. The nuclear expression of somatostatin receptors is scarcely described in the literature and the significance of this observation remains unclear. Perhaps a publication bias has affected this finding. Previously, Hornick *et al*. described a radioactive-labeled somatostatin analog uptake in the cell nucleus of human neuroblastoma cells (39). Some researchers have speculated that the antiproliferative and apoptotic properties of somatostatin are mediated through the protein 86-Ku, which has been shown to function as a somatostatin receptor. Binding of somatostatin to 86-Ku promotes the translocation of 86-Ku from the cytoplasm to the nucleus (40, 41). It is possible, however speculative, that the nuclear SST expression described here represents a cross-reaction between SST antibodies and 86-Ku.

No standardized scoring system for the immunohistochemical evaluation of SSTs currently exists. The scoring system used by Körner *et al*. (42) (examining SST_2_, in particular) yielded a strong correlation with the binding of the somatostatin receptor measured using *in vitro* receptor autoradiography. This was, however, primarily focused on membrane expression, which was marginally prevalent in our tumor samples. Specht *et al*. compared three different scoring systems on the SST expression in bronchopulmonary neoplasms: the immunoreactive score (IRS) originally developed for the evaluation of estrogen and progesterone receptors in breast cancer, the Her2/*neu* score developed for the evaluation of HER expression in breast cancer and the hormone receptor score (H score). These scoring systems were compared to the results obtained using real-time PCR, whereby all showed some correlation, although the IRS scoring system emerged with the strongest correlation to the RT-PCR results (43). All of these scoring systems rely on the percentage of stained cells in combination with the staining intensity. In our study, the intensity of the stained spots was generally weak and the percentage of stained cells was low, particularly regarding the nuclear expression. Analyses using either a higher cut-off point (counting weakly positive spots scored 1 as negative) or a minimum of 10% stained cells for positivity yielded insignificant results and the number of positive tumors decreased considerably. A cut-off point of 10% to be considered positive has been suggested for staining of SST_2_ (25); however, there is no consensus on the matter, as also a small number of receptor-positive tumor cells might be biologically and/or clinically relevant (42). A cut-off point as low as 1% has been used regarding other markers, such as PDL-1 (44). The actual or clinical significance of the weakly stained spots can be questioned. A higher staining dilution might further weed out false-positive spots. While the PPV for cytoplasmic SST_5_ is only 0.52, slightly better than chance, the NPV is 0.94. According to Juhlin *et al.*, the NPV of negative parafibromin staining is also around this magnitude (18). This suggests that a negative staining for cytoplasmic SST_5_ strongly indicates a benign tumor.

In general, SSTs represent potential targets for imaging and the treatment of NETs. Occasional case studies indicate that octreotide might affect PTH-associated hypercalcemia and reduce the urinary calcium output in primary hyperparathyroidism (45). For instance, Karacavus *et al*. published a case report of a SST_1_-positive parathyroid adenoma discovered on an octreotide scintigraphy (46). Long-acting octreotide has been proposed as a possible treatment for PHPT due to adenoma in MEN1 patients. Furthermore, somatostatin analogs have been tested in the treatment of hyperparathyroidism alongside surgery without an apparent effect on the patients’ serum calcium or PTH levels (20, 47, 48). Because PCs are so rare, parathyroid adenomas have primarily been used in these studies.

Among all SSTs, SST_2_ stands as the most commonly expressed throughout the body. In clinical settings, the most commonly used somatostatin analogs, such as octreotide and lanreotide, target SST_2_ in particular, and also share a certain affinity with SST_5_. Newer somatostatin analogs such as pasireotide and somatoprim have affinity for all receptor subtypes, although SST_4_ to a lesser extent (20). Presumably, membranous expression stands as a prerequisite for successful treatment or for imaging using octreotide or other somatostatin analogs. In our study, membrane expression was scarce and did not significantly differ between the tumor groups. However, the two SST_2_ and SST_3_ membrane-positive tumors were malignant; and we were particularly interested in these receptors due to the clinical significance of membrane positivity. We found that among the five patients who died of PC-related causes, one was SST_2_M positive and another was SST_3_M positive, indicating that these tumors might be more aggressive than their negative counterparts. New treatment options could have proved useful in these cases. Moreover, further research is necessary in order to conclude whether somatostatin analogs targeting these receptors could be used to treat membranous SST-positive PC tumors. In patients with metastatic disease not responding to conventional treatment, it may be possible to examine the expression of SST_2_M and SST_3_M for individually customized targeted treatment using somatostatin analogs.

PC is one of the rarest carcinomas known, making it difficult to study and treat. We studied a nationwide cohort (*n* = 32). The number of carcinomas studied was relatively small and our findings warrant validation in other cohorts. TMAs are not considered representative of the tissue as a whole. This was taken into account by taking several punches from each tumor, both from the middle and from the borders of the tumors. Our results are consistent, and our statistically significant findings follow a gradient with the highest SST expression in PCs without exception.

### Conclusions

Using immunohistochemistry, we demonstrate that SST_1–5_ are expressed in parathyroid tumors, either in the cytoplasm or the nucleus. The expression profiles of SST_2–5_ differ significantly between benign, atypical and malignant parathyroid tumors. Additionally, due to the large differences in expression between the tumor groups, SST_5_ represents a potential new immunohistochemistry panel marker for PC in addition to the established markers parafibromin and Ki-67 and suggested markers galectin 3 and PGP9.5.

## Declaration of interest

The authors declare that there is no conflict of interest that could be perceived as prejudicing the impartiality of the research reported.

## Funding

This work was financially supported by the Helsinki University Hospital Research Funds (TYH2017204, TYH2017138 and TYH2018223) and Finska Läkaresällskapet (to C Schalin-Jäntti).

## Author contribution statement

Study design: J A, C S J and C H. Study conduct: S S, H L, E R and J A. Literature review: S S, J A, C S J. Data collection: S S and E R. Statistical analysis: J L and S S. Drafting manuscript: S S, J A and C S J. Revising manuscript content: S S, H L, E R, J L, C H, C S J and J A. Approving final version: S S, H L, E R, J L, C H, C S J and J A.
